# Presidential Address : Postgraduate Medical Education

**Published:** 1925-01

**Authors:** J. Odery Symes

**Affiliations:** Senior Physician, Bristol General Hospital; Chairman of the Medical Postgraduate Committee, University of Bristol


### The Bristol Medico-Chirurgical Journal

"Scire est nescire, nisi id me
Scire alius scierit."

JANUARY, 1925.

### POSTGRADUATE MEDICAL EDUCATION.

The Presidential Address, delivered on October 8th, 1924, at the opening of the Fifty-second Session of the Bristol Medico-Chirurgical Society.

BY

J. ODERY SYMES, M.D., D.P.H.,

*Senior Physician, Bristol General Hospital;*
*Chairman of the Medical Postgraduate Committee, University of Bristol.*

THE initial, and indeed the most troublesome duty imposed upon the President of this Society is the selection of a subject for his inaugural address. The earlier Presidents chose some subject in medicine or surgery in which they were interested, or of which they had some special knowledge, and thereon based their discourse. More than one-half of our Presidents have followed this example. Dr. Lemuel Griffiths in 1887–88 made a new departure and gave a most delightful address on "Shakespeare and the Medical Sciences," and since that time there has been a succession of addresses of a literary or antiquarian character. We recall with great pleasure Dr. Rogers' address on "The Medical Aspect of Boswell's *Life*

*of Johnson,*" Dr. Parker's on the foundation of our Society, and Dr. Nixon's on " The Debt of Medicine to the Fine Arts." A review of medical practice and experience from a philosophic standpoint has been the foundation of several addresses. Most of us will recall Munro Smith's on " The Medical Life," followed next year (1903–4) by Paul Bush on " Some Surprises in my Professional Career." Samuel Martyn, who was elected President for 1876–77, died before giving his address from the chair, and in the following year Augustin Prichard, with a rare courage, for which I envy him, omitted altogether the Presidential duty. In 1912 Dr. Walter Swayne dealt with " The Problem of Medical Education," and the remarks which I purpose making to-night are supplementary to, and in a strict sense consequent upon, what he then said.

POSTGRADUATE MEDICAL EDUCATION.

It is my purpose to-night briefly to call attention to the inadequate way in which present-day methods of education fit us for general practice, and to inquire how far these deficiencies may be made good by the efforts of societies such as our own, and more particularly by systematic, organised, scientific, and clinical postgraduate medical teaching.

We shall, I am sure, all agree that the curriculum of the present-day student is overcrowded. The preliminary studies in biology, chemistry, and physics should be totally removed from the medical syllabus. They can be, and they are, taught during the school years, and should be included in any school leaving certificate or matriculation examination. I am not sufficiently in touch with the physiological and anatomical departments to criticise their methods, but most of us who are clinical teachers will agree that the practical side of these sciences is too often forgotten, and that the average student is taught as if he were to be a future professor of physiology or anatomy. The adaptation of these sciences to the needs of ordinary clinical work is quite lost sight of. If it were possible to classify undergraduate students according to their ability and future aims, it would result in a great saving of time for the average man, whilst at the same time better fitting him for the practical problems of medicine and surgery which he is about to meet in the wards. Our universities might then institute postgraduate courses in the preliminary sciences for those who are desirous of obtaining higher qualifications or becoming specialists.

Although three-fifths of our students are eventually to take up private practice, no effort is made to teach them even the rudiments of medical ethics or to instruct them in the many details that go to make up the successful art of visiting patients. The requirements of examining bodies, and consequently the schedule of university studies, make it imperative that the student should spend a large portion of his time in the study of a materia medica which is a hundred years behind the times, and of a pharmacology which has little relation to the needs of clinical practice. In consequence of this, the newly-fledged practitioner will find that just as his midwifery is handed over to nurses, and his fevers, tuberculosis, and venereal diseases to state and civic officials, so, owing to his unpreparedness, his work is gradually being encroached upon by the vaccinist, the psycho-therapist, the balnæologist, and the teacher of remedial exercises. It is, too, very largely due to the antiquated and very imperfect training given to our students that so many patients ultimately drift into the hands of the Christian scientist, the osteopath, and the cheiropractor. What we really want to do is to scrap entirely all our preconceived notions of what duties and subjects of study should occupy the hands and minds of our students during their phase of hospital attendance, and to begin afresh to apportion their time with a view to the requirements of modern practice and the newer methods of thought which animate the medical world to-day. I have no system of reform of medical education to bring forward, but I wish to emphasize that our profession entails *a lifelong course of study*, and the rapid progress of medical science makes it imperative that if a man is to preserve his intellectual freshness he must at least take what Osler has very aptly called " a quinquennial brain-dusting." It is my purpose to indicate to you in what way English-speaking peoples throughout the medical world are grappling with their professional educational deficiencies, and are striving by various methods of postgraduate study to keep abreast of the times, and to maintain the high standard of scientific and practical knowledge which has always characterised the medical profession.

### GREAT BRITAIN.

Turning our attention first to our own country. It is in London the most noteworthy effort has been made. The amalgamation of the Fellowship of Medicine with the Postgraduate Association was accomplished in 1919, and since that time postgraduate medical teaching has gone steadily forward. Nowhere else in the world is such a wealth of clinical material available. Some of the medical schools, the postgraduate college, and more than a score of general and special hospitals are available for intending pupils. Many of these hospitals admit only postgraduate students, and this is an enormous advantage to them, for it is most difficult to teach undergraduate and graduate students side by side. The courses roughly fall under two headings :—

(1) *Intensive General Courses* lasting two weeks, the object of which is to enable practitioners to brush up their general knowledge of medicine in its various branches, and

(2) *Special Courses*, which are designed to train our future specialists, or to advance the knowledge and technical skill of men in practice who wish to perfect themselves in any one particular branch of their art. The special courses have proved the more popular, and their duration varies from two weeks to a month. During the past twelve months special courses have been held in such subjects as Cardiology, Children's and Infants' Diseases, Fevers, Neurology, Gynæcology, Ophthalmology, Proctology, Psychology, Urology, and Venereal Diseases.

Year by year the London Postgraduate School is attracting more and more students, rather more than half of whom come from the Dominions, the United States, the Continent, Japan, and South America. The one great need appears to be a central building worthy to house this most important and rapidly-growing institution, one which can supply the classrooms and laboratories necessary for teaching purposes side by side with an adequate number of beds for general cases. Such provision must either be made by private munificence or by State aid—and once made, London will become, as it is fitted to become, the greatest centre of postgraduate teaching in the world.

The progress of medical postgraduate education in the *provinces* is a problem which presents peculiar difficulties of its own. Most of the universities provide postgraduate courses, which have met with more or less success.

At Manchester a regular course is given at each of the three General Hospitals and at one or two of the Special Hospitals. At the Manchester Royal Infirmary an advanced course (covering recent advances in medicine and surgery) has been well attended, and has been more than usually successful. The average attendance at Manchester has been 30 to 40 at the regular postgraduate lectures and 60 to 70 at the advanced ones.

At Sheffield regular postgraduate lectures have been given during the six months of the winter, as it was found difficult to secure attendances during the summer months. Two lectures per week are given for about five months.

At Liverpool lecture demonstrations for practitioners are given during the University terms; they are spread out over all the hospitals of the city, and are so planned that in any one day a demonstration is given in one hospital only—no fee is charged. The attendance is very variable, but it is found that many of the men are able to attend in the autumn term.

Birmingham has had postgraduate courses for the past twenty years, and seems to have met with considerable success. Last year the course extended over three months; the class has been held twice a week—for this there were 55 entries.

At Durham a general course of clinical demonstrations, meeting once a week, and spread over a period of three months, has met with a considerable degree of success, the attendance varying from 15 to 40. A vacation course lasting fourteen days, held in June, devoted to clinical lectures in general medical subjects, and post-mortem room demonstrations, was less successful. An interesting experiment was the provision of three months' courses in special subjects, such as Diseases of the Throat, Nose and Ear, Venereal Diseases, Gynæcology, and Diseases of the Skin. The meetings were held once a week, and were mainly clinical. A minimum of six persons formed a class, and the numbers have varied between this and eighteen.

In Scotland, postgraduate instruction at Edinburgh chiefly takes place during the summer vacation. Separate courses are given in Medicine, Surgery, Gynæcology, and Diseases of Children. In addition there are special courses in Diseases of the Blood and Vaccine Therapy, etc. A short course of eight to ten weeks on Diseases of the Nose, Ear and Larynx, particularly adapted for those who are training as specialists, is held during the autumn or spring terms. The Edinburgh postgraduate courses in Medicine are in connection with the University and the School of Medicine at the Royal Colleges, and they attract large numbers from all over the country and from abroad. Special lectures are held in the evenings at 5 o'clock, open to all local practitioners, in addition to those enrolled for the course; many of the local men attend. Altogether medical postgraduate work in Edinburgh seems to be well organised and worthy of this very distinguished and ancient medical school.

A very efficient course of study is provided by the Glasgow Postgraduate Medical Association in connection with the University of Glasgow. A series of weekly demonstrations given during the winter has been a most successful feature. Courses are also arranged during the summer months; these are of a whole-time nature, and are of about four weeks' duration. The Association publishes a syllabus of the various courses, and from this it is evident they are utilising all the resources of the *Special* Hospitals of the city; and in the General Hospitals vacancies are found for graduates who are willing to act as clinical assistants. There are also special courses on Genito-urinary Surgery, Medical Ophthalmology, Fractures, and Surgical Diseases of Children.

In Wales the distinctive feature of postgraduate Medical work is the course on Tuberculous Diseases conducted by the Welsh National School of Medicine for the diploma of the University of Wales.

All the provincial Universities find that courses consisting of one or two lectures a week are much better attended and more appreciated than intensive whole-time courses of one week to a month. Although here and there the attendance at postgraduate classes is fairly satisfactory, at nearly every centre one hears the same complaint, viz. that only a very small proportion of the medical men of the district are really anxious to take up any further course of study. The Secretary of one centre writes: "We find that the attendances on the whole are poor, and that a certain group of people—who really do not particularly require teaching—is the one which turns up time after time." Another writes: "Our attendance during the present term has dropped below ten. The Clinical Board feels that the lectures are well worth while if an average of ten is maintained; nevertheless ten out of a possible eleven hundred seems a very small number." From a third great provincial centre the Secretary writes: "We have met with considerable success in many ways, although the local practitioners do not avail themselves of the facilities offered as they should." There seems to be a very much greater keenness for postgraduate work in the North of England than elsewhere.

From a study of conditions in Bristol and the West of England, which one knows intimately, it is possible, I think, to visualise more accurately and minutely the extent to which postgraduate study is pursued throughout the medical profession. We have in the West a body of medical men of average keenness and average intelligence, living in a moderate-sized industrial town with fair facilities for post-graduate medical work, or residing in a country-side not too remote from educational centres. What steps does the average man take to keep abreast with the times, or up to date with his work? Undoubtedly the most widely-adopted and efficient measure is the taking of one or other of the medical journals—*The British Medical Journal, The Lancet, The Practitioner,* and our own *Bristol Medico-Chirurgical Journal.* Probably ninety per cent. of our profession read one or other of these journals, and their value in postgraduate teaching is extremely high. The small magazine published by the various big drug houses and the detached advertisements of smaller drug manufacturers are faithfully read by many, and unfortunately are often taken as the sole necessary education in modern therapeutics and pharmacology. The second most powerful factor in postgraduate education is undoubtedly the local medical society. The scientific meetings of the various branches of the British Medical Association in the West and throughout England are a potent factor in keeping alive our interest in professional work and keeping us informed as to new departures. In most moderate-sized towns there are also societies such as our own where men meet socially to talk the inevitable "shop," and where clinical cases are shown and papers are read. Our Society, with its meetings and journal, its reading-room and its library, is to my mind the most potent post-graduate educational force in the town. At Bath there is the Clinical Society, and farther West the Plymouth Medical Society has been holding meetings for a hundred and twenty-six years. The Devon and Exeter Medico-Chirurgical Society, the Honiton and Axminster Medical Society, the Torquay Medical Society, and many others are all doing good work, and represent what is happening in almost every big town in the kingdom. The number of men who attend such societies or use their magazines or libraries is, it is to be feared, comparatively small. From a study of attendance at our library and medical societies, I calculate that only between 15 and 20 per cent. of the profession ever avail themselves of the facilities offered. Valuable as is the teaching thus afforded, it is not systematic enough, nor is it as a rule sufficiently practical to meet the requirements of the busy practitioner, and there has been therefore in all countries a steady and increasing demand for systematised postgraduate teaching. In the University of Bristol this has been met in two ways :—

(1) Courses of lectures and demonstrations on general medical subjects have been held at the University, Hospital and Infirmary. These were given once a week during the summer months. The result was only moderately encouraging, the average attendance being about ten, and several of these were practitioners from the near country districts. An attempt to run a week of postgraduate study in some speciality (Ophthalmology) was even less successful, and had to be abandoned. Notices of these courses were sent to over five hundred doctors. (2) The second scheme, viz. the formation of classes in the smaller towns of the West of England, was much more successful, and for several years very well attended courses have been held in Trowbridge, Swindon, Barnstaple, Bournemouth, Dorchester and Hereford. In some cases these classes have been initiated by the local branch of the British Medical Association, and in others by private enterprise. The members of the class choose the subjects from a list sent by the University Director of Postgraduate Medical Studies, the lecturers all being members of the University staff. The meetings are held once a week for six or eight weeks, generally on the premises of the local hospital. The success of this experiment has excited much comment, and the procedure has been copied by other universities—one sees it referred to from time to time as "the Bristol Scheme."

Every year an increasing number of men apply for and are appointed to posts as clinical assistants at our principal hospitals, a most valuable method of postgraduate education. Immediately following the war there were a fair number of applications for refresher courses in general medicine, or in some speciality; such applications are, however, growing fewer, partly, I think, because it was found that the teacher was overwhelmed by the crowd of undergraduate students, only now just beginning to grow smaller. Other towns in the West of England have organised courses of postgraduate study independent of the University of Bristol. At Plymouth the staff of the South Devon Hospital, in conjunction with the local division of the British Medical Association, have organised very successful courses during the past two winters. The lectures were spread over a period of ten weeks, and about thirty medical men attended. It was largely due to the initiative and energy of the late Mr. Forbes Fraser that the postgraduate courses in Bath were so successful. The Bath United Hospital was the centre, and the exhibition of clinical cases was a most attractive feature. I am informed that the actual number of Bath practitioners who attended was small, but that men from the country districts readily joined, and attended regularly.

It is evident from what I have already told you that the facilities for postgraduate medical study in some form or other are generally available both in town and country, but I am not exaggerating when I state that not one man in ten ever avails himself of the opportunities offered. There are several possible explanations of this: (1) The teaching offered is not attractive enough. In some cases the lectures are too purely scientific, and have not sufficient bearing on the everyday requirements of the practitioner. This, I think, is not common, for as a rule men welcome any advanced work or theoretical ideas which stimulate their thought. Lectures on the highly technical work of advanced specialists sometimes prove boring. More commonly the lecturer under-estimates the intelligence of his audience. A postgraduate lecture must be carefully arranged and clearly delivered. It must be the lecturer's *best* effort, and must serve at least two purposes, viz. the presentation of recent advances in the subject, and the useful adaptation of modern work and research to everyday practice. (2) Inability to spare the time and expense which a postgraduate course entails is a common excuse for non-attendance. Many a man explains that he cannot afford to have both a holiday and a fortnight's refresher course, and he must have a holiday. At a time like the present, when the remuneration of the general practitioner is higher than it ever has been, the small fees which are charged for postgraduate courses are negligible. If a period of absence cannot be obtained, there can be but few men, other than those living in remote country districts, who cannot spare two or three hours on one day a week to attend some centre of instruction. The proof of this lies in the fact that here in Bristol, and in almost every provincial centre with which I have been in communication, it is found that the first men to join and the most regular men to attend are the practitioners from outlying country districts, who must be sacrificing at least half a day. The fact of the matter is that city practitioners do not find it necessary to keep themselves so fully up to date, or so practically efficient, as their rural brethren. In any emergency they can call in a fellow practitioner or a specialist, or seek the welcome oblivion of a hospital. I think you will one and all agree with me that one of the dangers of the Panel system in big towns is that scientific and practical interest in medicine may decline, and professional work become more and more commercialised. (3) Another objection to attendance at postgraduate courses which we have met with is, that if it were known that a man were doing such a course, it would be regarded by his patients as a reflection on his competence and knowledge. Such a view, however, could only be held by the more ignorant, and in any case it is necessary to educate public opinion in this matter. More and more medicine is becoming a State service, and I hope that the day is not far distant when no man or woman will be allowed to hold any post under State or Municipality, in the Services, or on the Panel, without producing at certain stated intervals a certificate showing that he has satisfactorily attended some approved postgraduate course; such a course should be provided free of cost and whilst on full pay. (4) A very real difficulty in medium-sized towns in organising postgraduate work is that men are unwilling to attend lectures given by those in daily association and competition with them, and they also fear to import lecturers from neighbouring university centres, lest in doing so they should introduce to the district consultants who might attract work which is at present done locally. This difficulty, which is a real one, can only be overcome by a frank recognition of the fact that every man has something to contribute to the common stock of knowledge, and that a postgraduate clinique is not so much a place where one is teacher and the rest students, but rather a meeting of medical men on an equal footing to discuss and review points of common interest. The area served by consultants from a university centre is determined chiefly by geographical factors, and it is very doubtful whether the delivery of a postgraduate lecture is likely to extend the boundary. Indeed, the lecturer may, if he be not acceptable, very considerably curtail it. On the other hand, it is quite certain that wherever postgraduate centres have been formed the localities have tended to become more self-sufficing, and as the custom grows we shall more and more see country centres becoming increasingly independent of both the consulting physician and the operating surgeon. Everything that tends to raise the level of medical education in an area tends not only to increase the interest in the work but also to raise the standard of its remuneration.

### UNITED STATES.

I think I have said enough to show you that although there is in this country a growing demand for postgraduate medical education, yet this demand is a limited one. We are not doing our best in this matter, and I doubt whether the bulk of the profession is as keenly interested in the clinical and scientific sides of medicine as they were, say, twenty years ago. We do not compare favourably with our American brethren, for instance. From letters I have received, and from other sources of information, I gather that in the United States the general practitioner is closely associated with hospital work, he expects to have periodical times of study leave, and his patients realise that such periods of absence are necessary and are for their benefit. At the Annual Congress on Medical Education, Medical Licensure, Public Health and Hospitals, held in Chicago in March, 1923, the total number of graduate students enrolled in 1922 was stated to be 3,068. The matter has been well summarised in the report of an inspection of all graduate medical schools in the United States, made by the Secretary of the Council of the American Medical Association and Dr. Louis B. Wilson of the Mayo Foundation, in October, 1922. They note that there is a decided improvement in the character of the graduate medical schools since the survey in 1919. They are satisfied that whilst thorough work on the highest plane of university graduate study is in progress, yet the development of opportunities for graduate study is still far from adequate. Work on the lines of the Bristol scheme is growing, and increasing opportunities are afforded to county, state, and district medical societies to organise and operate once or twice each year diagnostic clinics, as well as extension courses of lectures and clinics given under the auspices of the Universities of North Carolina, Pennsylvania, Wisconsin, and Washington, and by several of the state medical societies and state university medical schools. Opportunities for postgraduate study in pre-clinical branches are afforded by several university medical schools to a greater extent than in this country, but there has been no material increase in the number of students availing themselves of such opportunities. Postgraduate students in the States fall under one of two heads: (1) those preparing themselves for the practice of some speciality, (2) those desiring to improve themselves in general practice. The conditions governing specialists have been much the same as in this country, that is to say, the future specialist prepares himself by holding a resident post, clinical clerkship, or assistantship in some special clinic, and by self-proclamation determines when he is fit to practice, his qualification being that he pays a subscription to some association or society of specialists. Two universities, however (Minnesota and Pennsylvania), have well-organised courses for the efficient teaching of pre-clinical and clinical work in various specialities, and give degrees of Master of Science or Doctorates in the speciality selected. We have, of course, in England M.D. degrees and various diplomas in special subjects, but our difficulty is to obtain the special postgraduate laboratory and clinical teaching to qualify for them.

The second class of students, viz. those seeking refresher courses, are well provided for by lecture demonstrations and laboratory work; but as is pointed out, the courses are so brief that the student cannot be entrusted with full responsibility for the cases. Such courses are, I notice, generally longer and the fees are higher than in this country. For instance, the courses given for qualified graduates in medicine by the Columbia University extend over six weeks, and the fee is 200 dollars, and this fairly represents the conditions elsewhere.

The principles suggested for grading postgraduate medical schools in America are, I think, of some interest, and would apply equally well to this country. The report suggests that candidates wishing to qualify as specialists must not only graduate from a Class A medical college, but shall have completed at least one year's internship in an approved hospital. The previous records of intending students must be sent in, so that the character and grade of the work may be rightly assigned. The college in which they work should provide review courses in the pre-clinical sciences which apply to the respective specialities ; clinics for the treatment of both in- and out-patients ; courses of operative and laboratory technique ; opportunities for taking the sole care of patients, and of pursuing research work in the speciality. Emphasis is laid on the fact that for refresher courses it is not sufficient to give lectures only, there must be ample clinical material such as can be provided by a hospital of at least 200 beds, and with an out-patient attendance of at least 100 patients a day. Library and museum facilities must be available, and all teachers must be of a high grade.

The distinctive features of American postgraduate education are as follows : It is almost entirely under the direction and control of university authorities, and this more than anything else gives it an added strength and prestige. It is realised throughout the country that postgraduate education is as important as undergraduate education ; and through the generosity of private benefactors postgraduate medical study is very largely provided with separate hospitals, laboratories and teaching quarters. In the United States the aim is more particularly the training of the specialist by a course lasting for two or three years, and at the end of that period a distinguishing degree is granted. It is recognised that a man so trained is an asset to the community, and he is therefore given pecuniary help in the form of scholarship or fellowship during his second and third years of study. Refresher courses are made to extend over a period averaging about six weeks, and the courses of lectures given at outlying centres are more highly organised than in this country, and the clinical material of the local hospitals is better utilised, the visiting lecturer finding the patients and records awaiting his arrival, and being afforded time to study these before the conference begins. The number of teachers engaged is much larger than in this country, and it is recognised that their work must be adequately paid; also wider use is made of the system of intern appointments after qualification.

It would appear that the difficulty in America is that there is a very wide variation in the standard of graduates, and this is being met either by insisting that the candidates for postgraduate degrees must already hold a diploma of a Class A medical school, or by grading the candidates when they present themselves for registration, and then grading their work or insisting on a longer period of study.

It is not possible for me to enter into any detail about the courses provided by the various American colleges, but there are some with special features which I should like to mention. The New York Postgraduate Medical School has its hospital of 400 beds, an out-patient department with 800–1,000 patients a day, and a separate school of nursing. For forty years it has been engaged in teaching physicians, and there can be few institutions better equipped for the work. Columbia University affords most excellent opportunities for postgraduate study, and during the past year 160 graduate students attended. Columbia divides the workers into two divisions; the first are doing original investigation and have one year in residence, and the second are engaged in continuous education on the level of ordinary university extension work. The Graduate School of Yale University gives a doctorate in Public Health, a doctorate of Philosophy, and a Master of Science degree to men holding the M.B. of accepted standard. They require a term of residence of either two or three years, during which time a definite organised and formalised course is pursued. In addition to courses on general medical subjects, Harvard seems to afford special facilities for the pursuit of pre-clinical studies,

Anatomy, Physiology, and Biological Chemistry. Cornell and the Graduate School of Wisconsin University similarly have excellent classes on the scientific subjects, including opportunity for general and research work in Pharmacology and Toxicology. We have too few opportunities for such study in this country. The University of Pennsylvania affords very good facilities for student physicians, having access to some fifty institutions in Philadelphia. I notice one paragraph in the report for 1923–24 which is calculated to open our eyes: "Of the University's assets, $2,500,000 is the amount devoted exclusively to the Graduate School of Medicine; and the School has the advantage of participation, according to its needs, in the use of further University facilities to the value of at least $2,000,000."

Contrast this with the conditions in Bristol and elsewhere in Great Britain, where postgraduate education is practically an unpaid service supported by the public spirit of the medical profession. Pennsylvania grants certificates and degrees to successful regular student physicians. Such certificates, which must be clearly distinguished from diplomas and degrees, ought to be more generally given in this country and by our own University. They state the date of registration, the attendance, and the nature of work accomplished. The degree of Master of Medical Science is conferred after successful completion of a second year, and the degrees of Doctor of Medical Science upon completion of the third year of a regular course. There is a generous provision of scholarships and fellowships during these two years. The Rush Medical College of the University of Chicago, in addition to their graduate courses in Medicine and Preliminary Sciences for Physicians, have a somewhat new departure for those wishing to take up a speciality. In Otology and Laryngology there is to be a one year's full-time course, half of each day being spent in the study of the fundamental sciences and the other half in the clinical study of patients. Only eight students are taken each year, two entering each quarter. The Mayo Foundation, now a part of the Medical School of the University of Minnesota, is a very active body ; a thousand candidates a year apply for graduate medical training, and about 200 a year complete their applications. The foundation has concentrated very largely on the production of specialists by a system of fellowships. The fellowships entail three years' graduate study and residence, in preparation for either the special degree of Master of Science or Doctor of Philosophy in Medicine, Surgery and Pathology, etc. Postgraduate work at Johns Hopkins University is limited at present, owing to structural alterations, to one course of six weeks' duration for general practitioners. At present the tide of students flows steadily from America to Europe, but the organisation of American Postgraduate Schools is so excellent, the equipment so complete, the financial support so generous, and the standard of study so steadily improving, that I venture to predict with confidence that in the next ten years the tide will be reversed, and we shall see an increasing number of postgraduate medical students from all nations seeking the refreshing courses of American medicine.

### THE DOMINIONS.

I do not propose saying very much about the progress of postgraduate study in the Dominions, but would wish to draw your attention to a scheme very like our own (Bristol) scheme which has had a phenomenal success in Canada. The Ontario Medical Association, with the co-operation of the Medical Faculties of Queen's and Western Universities, and the University of Toronto, has been organising postgraduate work throughout the province through the county and local medical societies. In this they have had the co-operation of the Department of Health of Ontario, and the generous annual contribution of $5,000 from the Ontario Red Cross Society. The work consists either of individual lectures, forming part of local society meetings, or of individual lectures to groups of members of county societies, who wish for special instruction, or of three or more lectures, properly correlated as to material and in proper sequence, making up a series. About 250 lectures are given a year. A schedule giving the titles of some 300 lectures is sent to each society, and when the choice of lecture is made, the name of the lecturer is given. Professor Austin (who is known to many of you) may indeed write that this is "A most up-to-date and progressive system. . . . the most complete in any province or state of Canada or the United States." The University of Manitoba gives a three weeks' intensive course for graduates usually at the close of the regular summer session. During the past two years the average attendance has been about twenty, which we should consider good in this country.

Professor Ferguson of the University of Otago, New Zealand, tells me that short postgraduate courses have not met with much success, as it is difficult for men in practice to obtain *locum tenens*. He states: "Our practitioners in New Zealand prefer to take longer spells than a short post-graduate course implies. Instead of coming to get rubbed up in their work every two or three years, they wait six or seven years, and then go home to Great Britain for six months' work there, or for six months' holiday with a certain amount of work thrown in. Some of them have taken regular classes at the Polyclinic or at one of the London hospitals, but of late there is a great tendency for them to go to America and spend their time in the clinics there. I think those who go to America do more solid work during their trip than those who go home. A visit to the Old Country has distractions in the way of friends to be visited and old ties to be renewed, while those who go to America go to some particular centre for the sole purpose of studying the methods of some particular man."

Had time permitted I should have liked to tell you of the success of postgraduate medical work in Australia. There are courses at Sydney and Adelaide, both under University direction. In Victoria the arrangement of postgraduate courses is in the hands of a permanent Postgraduate Committee elected by the lecturers themselves, two representatives being sent from the Victorian branch of the B.M.A., and one from each of the following: the Faculty of Medicine of the University; one from each of the teaching hospitals; and the Director of the Hall Research Institute. For the fortnightly revision course the average attendance is 40 to 50; for the weekly winter evening lecture course 60 to 100. Much good work is also done by sending lecturers to such divisions of the B.M.A. as ask for their services at their quarterly meetings. Such enthusiastic support is given to the work that my correspondent is well justified in saying: "Throughout Australia the most gratifying response has been made to postgraduate work both on the part of those giving lectures and those attending them, and it is felt that this will increase as time goes on until all medical practitioners become accustomed to postgraduate instruction as an essential part of their training."

I think I have said enough to show you that there is at the present time in all English-speaking countries an awakening of interest in postgraduate study.

I must apologise for boring you with so big a mass of detail, but my desire has been that in viewing the progress made at so many and so important centres, we may be spurred on to further efforts ourselves. I am not referring now to the University, or to individuals, but more particularly to this great postgraduate body, the Bristol Medico-Chirurgical

Society. Starting afresh after fifty years of usefulness, in new premises, with added facilities for study, it is our duty, and it should be our pleasure, to do something to enhance the prestige and increase the usefulness of the Society. All can contribute to this end — by regular attendance at meetings, by enlisting new members, by contributing pathological material, papers, or cases for discussion, or by studying beforehand the subjects that are to come up at the meeting and preparing something to say. Those of you who have read Osler's *Æquanimitas* will remember the remarkable essay " On the Educational Value of the Medical Society." He says : " The doctor's postgraduate education comes from patients, from books and journals, and from societies, which should be supplemented every five or six years by a return to a postgraduate school to get rid of an almost inevitable slovenliness in methods of work." He then goes on to describe how the medical society lays a foundation for that *unity* and *fellowship* which is essential to the dignity of the profession ; how it should represent a *clearing-house* where each can receive his intellectual rating, and find out his professional assets and liabilities ; but its chief end and object must be to maintain and foster amongst its members a high standard of learning and a progressive knowledge of their professional work. Such ideals have guided the work of your Society in the past, and I cannot do better than close with Osler's words : " In the dedication of his *Holy War*, Thomas Fuller has some very happy and characteristic remarks on the bounden duty of a man to better his heritage of birth or fortune, and what the father found glass and made crystal, he urges the son to find crystal and make pearl. Your heritage has been most exceptional, and, I believe, from all that I know of the profession in this city . . . that could your fathers return they would say that of their crystal you had made pearl."

